# Simulation for teaching cardiorespiratory resuscitation by teams: setting and performance assessment*

**DOI:** 10.1590/1518-8345.3932.3406

**Published:** 2021-07-02

**Authors:** Emílio Carlos Alves dos Santos, Cor Jesus Fernandes Fontes, Eloana Ferreira D’Artibale, Jocilene de Carvalho Miravete, Gimerson Erick Ferreira, Mara Regina Rosa Ribeiro

**Affiliations:** 1 Universidade Federal de Mato Grosso, Hospital Universitário Júlio Muller, Cuiabá, MT, Brazil.; 2 Universidade Federal de Mato Grosso, Faculdade de Medicina, Cuiabá, MT, Brazil.; 3 Universidade Federal de Mato Grosso, Faculdade de Enfermagem, Cuiabá, MT, Brazil.

**Keywords:** High Fidelity Simulation Training, Professional Education, Cardiopulmonary Resuscitation, Simulation Training, Continuing Education, Cardiopulmonary Arrest, Enseñanza Mediante Simulación de Alta Fidelidad, Educación Profesional, Reanimación Cardiopulmonar, Entrenamiento Simulado, Educación Continua, Arresto Cardiopulmonar, Treinamento com Simulação de Alta Fidelidade, Educação Profissionalizante, Reanimação Cardiopulmonar, Treinamento por Simulação, Educação Continuada, Parada Cardiopulmonar

## Abstract

**Objective:**

to evaluate the acquisition of cognitive knowledge in cardiorespiratory resuscitation through training mediated by health simulation and to verify satisfaction with the teaching methodology design.

**Method:**

a study of quasi-experimental intervention, of the before and after type, with only one group. Population composed of medical students in the internship phase, nurses and resident physicians, nursing technicians and nurses of the institution’s effective staff. Convenience sampling consisting of 91 participants. Data collected through the Sociodemographic and Educational Questionnaire, Knowledge Test and Simulation Design Scale. Data was organized in tables and analyzed based on absolute frequencies, measures of central tendency and dispersion, Cronbach’s alpha reliability test, Wilcoxon’s test.

**Results:**

the increase in cognitive learning was 81.9%, being that for nursing technicians it was 117.8 %. Wilcoxon’s test showed a significant increase (p<0.0001) in knowledge. The Simulation Design Scale, displayed 4.55 of global mean. Cronbach’s alpha pointed good internal consistency (0.898).

**Conclusion:**

the health simulation was effective as a learning-teaching method in cardiorespiratory resuscitation, being effective in increasing knowledge in cardiorespiratory arrest, with a great level of design satisfaction.

## Abstract (es)

**Objetivo:**

evaluar la adquisición de conocimiento cognitivo en reanimación cardiorrespiratoria a través del entrenamiento mediado por simulación de salud y verificar la satisfacción con el diseño de la metodología de enseñanza.

**Método:**

estudio de intervención cuasiexperimental, del tipo antes y después, con un único grupo. Población compuesta por estudiantes de medicina en etapa de prácticas, enfermeros y médicos residentes, técnicos de enfermería y enfermeros del personal permanente de la institución. Muestreo de conveniencia compuesto por 91 participantes. Datos recolectados a través del Cuestionario Sociodemográfico y Educativo, prueba de conocimientos y Escala de Diseño de Simulación. Los datos fueron organizados en tablas y analizados con base en frecuencias absolutas, medidas de tendencia central y dispersión, prueba de confiabilidad alfa de Cronbach, y prueba de Wilcoxon.

**Resultados:**

el aumento del aprendizaje cognitivo fue del 81,9% y para los técnicos de enfermería fue del 117,8%. La prueba de Wilcoxon mostró un aumento significativo (p<0,0001) en el conocimiento. La Escala de Diseño de Simulación presentó una media global de 4,55. El alfa de Cronbach mostró buena consistencia interna (0,898).

**Conclusión:**

la simulación de salud resultó eficaz como método de enseñanza-aprendizaje en reanimación cardiorrespiratoria, siendo eficaz para incrementar el conocimiento en parada cardiorrespiratoria, con un excelente nivel de satisfacción con respecto al diseño.

## Abstract (pt)

**Objetivo:**

avaliar a aquisição de conhecimento cognitivo em reanimação cardiorrespiratória por capacitação mediada por simulação em saúde e verificar a satisfação com o *design* da metodologia de ensino.

**Método:**

estudo de intervenção quase-experimental, do tipo antes-depois, com único grupo. População composta por estudantes de medicina em fase de internato, enfermeiros e médicos residentes, técnicos de enfermagem e enfermeiros do quadro efetivo da instituição. Amostragem por conveniência formada por 91 participantes. Dados coletados por meio do Questionário Sociodemográfico e Educacional, teste de conhecimento e Escala do *Design* da Simulação. Os dados foram organizados em tabelas e analisados com base em frequências absolutas, medidas de tendência central e dispersão, teste de confiabilidade alfa de Cronbach, teste de Wilcoxon.

**Resultados:**

o incremento de aprendizagem cognitiva foi de 81,9%, sendo que para técnicos de enfermagem foi de 117,8 %. Teste de Wilcoxon apontou incremento significativo (p<0,0001) no conhecimento. A Escala do *Design* da Simulação, apresentou 4,55 de média global. Alpha de Cronbach apontou boa consistência interna (0,898).

**Conclusão:**

a simulação em saúde foi eficaz como método de ensino-aprendizagem em reanimação cardiorrespiratória, sendo efetiva no incremento de conhecimento em parada cardiorrespiratória, com ótimo nível de satisfação quanto ao *design*.

## Introduction

Health Simulation (HS) is an effective teaching-learning methodology for the development and acquisition of skills and abilities, contributing to qualifying patient care^1^. In the health context, it is defined as the insertion of the student or professional in a simulated environment that imitates some clinical aspect of their reality^2^. 

The efficacy of a HS with production of benefits requires systematic actions, with well defined stages, clear objectives, prior planning, trained facilitators, and availability of material and technological resources^3^. 

The experience of participation in HS is directly associated with the quality of the setting and the factors involved, such as the level of fidelity of the simulators, which can be of high, medium and low fidelity, as well as the credibility of the case elaborated^4^. In this sense, the construction of settings for simulation must go through three stages, namely: construction, application and assessment^3^. The same author reports that there is growing concern about the stage of building settings for simulation, since many institutions lack or do not have tools to support this moment, which may compromise their effectiveness. 

We understand the design in the context of health simulation as the structure adopted for its elaboration, in which a roadmap should be followed in the construction, as oriented by the good practices in simulation^5^^-^ 6. The application of the setting comprises three moments, briefing, intrassimulation, and debriefing. The first one is defined as the instruction moment of the case to be solved. Intrassimulation consists in implementing the proposed simulation. The debriefing is the moment when the participants reflect on their experiences and feelings, articulate theory and practice through critical thinking, and discuss aspects of simulation relevant to their practice, among others^6^. 

Setting assessment is an important stage, and should be applied continuously, based on the understanding that all settings need constant adaptation, even having been tested before^6^. The strengthening of this teaching-learning strategy can be optimized with the use of instruments that allow for its assessment, and thus assertively understand how HS participants assimilate it^3^. 

The fact that there are few training proposals in the area of cardiorespiratory resuscitation, with the use of HS in the study region, reinforces the importance of the research. Cardiorespiratory resuscitation (CRR) is one of the possible competencies inherent to health professionals that can be developed and improved by simulation settings^7^. 

In most of the studies found in the scientific literature, the most intense use of simulation in the training of health professionals is noted and few works using this methodology for the training of health professionals in the work environment. In this sense, national and international studies were found describing the use of simulation in graduation for physicians^8^^-^ 10 nurses^11^^-^ 16, as well as for nursing technicians^17^. So far, no Brazilian studies have been published on training mediated by simulation with a multi-professional team in cardiorespiratory arrest (CRA) care. Thus, in accordance with the recommendation of the American Heart Association (AHA), that the advanced life support be applied by multi-professional teams^18^, and in view of the scarcity of production, it becomes relevant and necessary to carry out studies with this scope to elucidate aspects not yet disclosed in this type of intervention.

Moreover, since it is a teaching hospital (TH), the insertion of the academic community is relevant, due to this being the real profile of the teams that attend daily CRA clinical situations. 

For this reason, this study was designed to evaluate the acquisition of cognitive knowledge and satisfaction with the teaching methodology by health professionals and students in the process of the teaching-service integration, submitted to training in basic life support (BLS) and advanced life support in cardiology (ALSC) mediated by the HS.

## Method

This is a quasi-experimental interventional study, of the before and after type, with a single group, carried out at the Júlio Müller University Hospital Júlio Müller (Hospital Universitário Júlio Müller, HUJM), of the Federal University of Mato Grosso (Universidade Federal de Mato Grosso, UFMT), located in Cuiabá-MT, from March to June 2018. The HUJM is a medium-sized general hospital, with one hundred and eighteen (118) beds, of which eighteen (18) are for adult and neonatal intensive care.

For a non-probabilistic convenience sample, the following inclusion criteria were defined: being a physician, nurse or nursing technician at the institution, being a physician or nurse in the institution’s internship program, being a medical student in the internship phase. All the participants who were invited to participate in the study accepted. However, at the time of data collection, those who did not complete all the stages provided for in the intervention, or who did not complete the data collection instruments were excluded from the study. 

Two instruments were used to collect data: the first one, a questionnaire to characterize the participants, containing sociodemographic and educational variables, including age, gender, training institution, participation in curricular or extracurricular activities related to the theme; the second one, a questionnaire built by the authors, composed of 27 objective multiple-choice questions, elaborated based on the 2015 AHA guidelines, to assess knowledge about the protocol for adult CRA care, addressed the following data: recognition of a CRA, CRR sequence in BLS and ALSC, chest compression technique, airway handling and manual external defibrillator, indication of defibrillation based on heart rate, drug administration in CRA and post-CRA care; the third one, Design Scale of the Simulation (Escala do Design da Simulação, EDS), developed by the National League for Nursing (NLN), used to evaluate the structuring of HS settings, in the participants’ perception. Translated and validated in a national study^3^, the EDS is made up by twenty items, distributed in five factors (objectives and information, support, problem resolution, feedback/reflexion, and realism). The internal consistency for the answers to the factors of the EDS instrument was high (Cronbach’s alpha = 0.898). 

**Figure 1:**
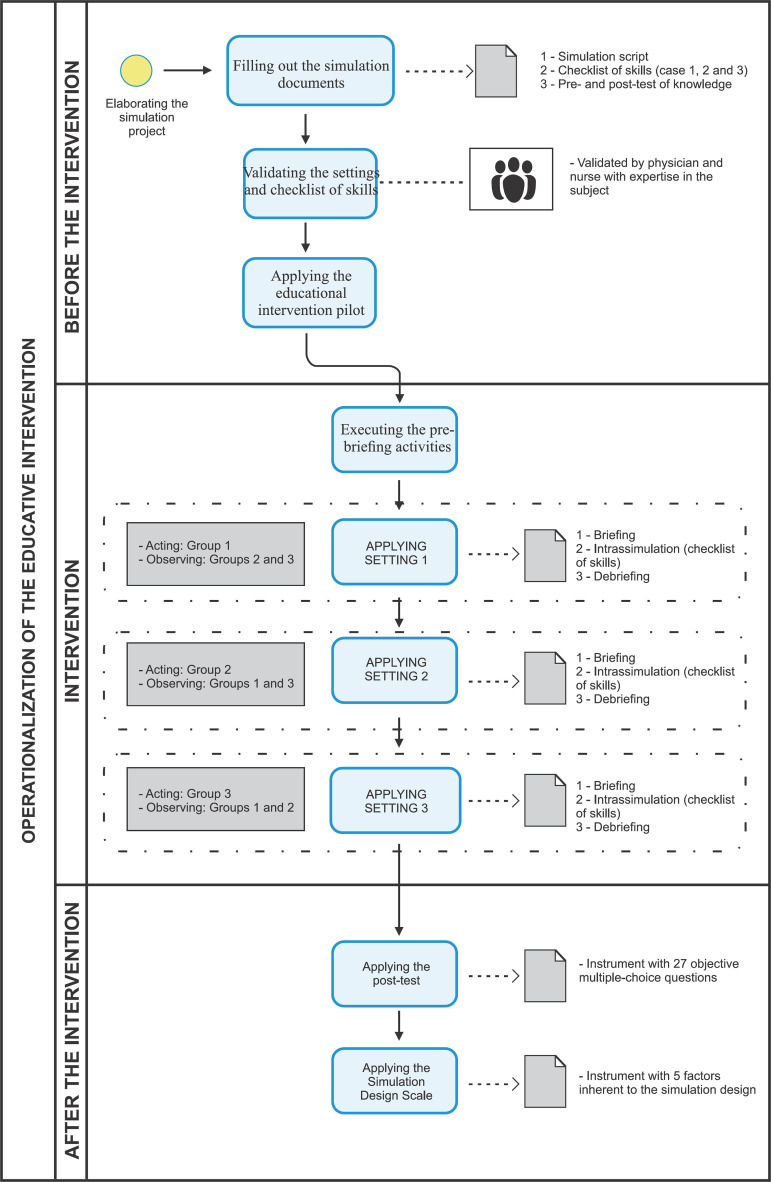
Flowchart for the operationalization of the educational intervention

Within this scope, an educational activity on cardiorespiratory resuscitation in adults was developed, using the HS for implementing data collection. Collection took place at two different times, with teams of exclusive collaborators intended for their application. The collaborators were previously trained and based their actions on the standard protocol for each phase of the study (Figure 1). 

In the first moment of the collection, the participants were informed about the study, especially on the educational intervention, invited to take part in the research, and expressed their acceptance by signing the Free and Informed Consent Form (FICF). The Sociodemographic and Educational Questionnaire and the Pre-Test of Knowledge were applied after the consent. After the theoretical lesson on the main AHA updates of 2015, the participants were divided into three groups and went through three skill stations, namely: primary assessment, airway management and electro-therapy.

In the afternoon period before the simulations, a brief expository lecture was given about effective communication and application of monitored practice in a CRA situation. Then, the participants were divided into three groups, so that each group participated in a different setting, involving the care of adult patients affected by CRA. After all the stages of the intervention have been completed, the participants were gathered in an auditorium and again answered the questions applied in the Pre-Test of Knowledge and then the EDS. 

The settings took place in a controlled environment - clinically adapted simulation room, containing: control room, partition with unidirectional mirror, cameras and microphones installed in the ceiling; equipment and materials - emergency car, CMOSDRAKE Life 400 Plus^®^ manual external defibrillator cardioversor, automated hospital bed, low- (RessusciAnne^®^) and high-fidelity (DartSim^®^) simulators.

The analysis of the results of this study focused, therefore, on the data obtained from the sociodemographic and educational profile, the evolution of knowledge after HS, by means of pre- and post-assessments and of the ESD. Data was initially structured in a Microsoft Excel spreadsheet and the typing was performed by two research assistants, with a subsequent check performed by the researchers. Then, the data were exported to the Statistical Package for the Social Sciences Version 23.0 (SPSS version 23.0) software. 

The difference in the participants’ learning about the CRR in the HS was evaluated by comparing the points obtained in the pre- and post-assessments, using Wilcoxon’s statistical test for paired data, with an α error of 0.05, assuming no normality for the data.

The project was approved by the Research Ethics Committee of the Júlio Müller University Hospital (Comitê de Ética em Pesquisa do Hospital Universitário Júlio Müller, CEP-HUJM), under opinion No. 2,441,281 and CAAE record: 80249217.0.0000.5541, based on Resolution No. 466, of December 12th, 2012.

## Results

Of the 91 participants, 70 (76.9%) were health professionals, among which 40 were nursing technicians, 29 were nurses, 01 was a physician and 21 (23.1%) were medical students, who worked in different sectors of the hospital and in more than one daily work shift. As for the institutional bond, 64.8%(n=59) were professionals in the hospital staff. More than 60% (n=57) were women and their age ranged from 22 to 55 years old, with a mean (SD) of 34.2 (8.0) years old, with a predominance of individuals between 22 to 50 years old. Regarding the level of schooling, 59.3% of participants had complete higher education. The public institution was responsible for training 50 (54.9%) of the participants. No graduate studies were reported by 70.3% of the participants and, of those who attended, the majority (23.1%) reported some specialization, that is, *lato sensu*. As for previous experience or training in CRR and health simulation, it was found that 50.5% participated in an extension course in basic or advanced life support, 78% had direct experience in CRA and only 5.5% in accredited support courses of life, and 18.7% participated in activities using the simulation and health teaching methodology.

Table 1 shows the descriptive analysis of the result of the cognitive pre- and post- assessment. The mean (SD) for the number of correct answers in the pre-assessment was 11.8 (4.95) and, in the post-assessment, 18.0 (4.37). Based on the visualization of the graph and the comparative analysis of the scores obtained in the pre- and post-assessment, it was evident that there was a significant increase (p<0.0001) in the participants’ knowledge about the CRR with the HS. The overall percentage of improvement in post-assessment performance was 81.9% (n=91).

**Table 1:** Distribution of the number of participants (n=91), with scores lower and higher than 70%, comparison test between the correct answers scores in the pre- and post-tests, by category. Cuiabá, MT, Brazil, 2018

	Functional category	<70% of correct answers	> = 70% of correct answers	Mean (%)	SD*	MIN^†^	MAX^‡^	% of improve-ment^§^	p^||^
General (n=91)									
	Pre	81(89.0)	10(11.0)	11.8 (43.7)	4.95	1	22	81.9	<0.0001
	Post	41(45.0)	50(55.0)	18.0 (66.7)	4.37	7	24		
Nursing technician (n=40)									
	Pre	40 (100.0)	0	8.4 (31.2)	4.04	1	18	117.8	<0.0001
	Post	31(77.5)	9(22.5)	14.9 (55.1)	4.22	7	22		
Medical intern (n=21)									
	Pre	14(67.0)	7(33.0)	14.7 (54.5)	4.36	8	21	55.9	0.0001
	Post	2(9.5)	19(90.5)	21.0 (77.6)	2.54	12	24		
Nurse (n=19)									
	Pre	17(89.5)	2(10.5)	14.2 (52.2)	3.51	7	19	50.1	0.0002
	Post	6(31.6)	13(68.4)	19.9 (74.1)	2.62	15	24		
Resident (Nur^¶^, Phy**) (n=11)									
	Pre	10(90.9)	1(9.1)	14.4 (53.5)	4.16	7	22	56	0,005
	Post	2(18.2)	9(81.8)	20.5 (75.4)	2.48	15	23		

* SD = Standard deviation;

† MIN = Minimum;

‡ MAX = Maximum;

§ Percentage of participants who improved their performance after the training. There may have been great null difference, that is, the nurses continued to answer the same question after the training;

|| Wilconxon's test;

¶ Nur = Nurse;

** Phy = Physician

Among the results of the knowledge assessment, it can be seen in Table 1 that ten (11.0%) participants obtained correct answers above 70% in the pre-assessment. After the intervention mediated by simulation, this number increased to 50 (55.0%). In the analysis by professional category, with the exception of nursing technicians, more than half of the other participants/category had correct answers above 70%. 

In general, in the acquisition of knowledge regarding the recognition of CRA, there was a mean of 53.8 points of correctness (Table 2). The overall mean of the post-assessment was 60.4. The critical item related to the indication of defibrillation based on rhythms was that of the lowest percentage (32.6%) of correct answers. The critical item with the highest success rate in the post-assessment was that inherent to the CRR sequence in BLS (84.1%).

**Table 2:** Mean percentage of hits in the pre- and post-assessment, according to the critical items evaluated. Cuiabá, MT, Brazil, 2018

Variables	General		TE*		INT^†^		ENF^‡^		RES^§^	
	Pre	Post	Pre	Post	Pre	Post	Pre	Post	Pre	Post
CRA recognition^||^	53.8	60.4	43.8	45	61.9	83.3	60.5	63.2	63.6	68.2
CRR sequence^¶^ in BLS**	47.3	84.1	36.3	73.8	45.2	88.1	60.5	94.7	68.2	95.5
Chest compression technique	48.4	72.9	32.5	61.7	60.3	82.5	56.1	77.2	69.7	87.9
Airway management in CRA^||^ (BLS** and ALSC^††^)	34.1	55.3	29.2	48.3	39.7	63.5	40.4	61.4	30.3	54.5
CRA care sequence^||^ in ALSC^††^	55.3	76.2	40.8	59.2	61.9	93.7	63.2	86	81.8	87.9
Handling the manual external defibrillator	44.4	51.6	38	45	46.7	60	50.5	54.7	52.7	54.5
Defibrillation indication based on cardiac rhythms	32.6	67.4	12.5	44.2	65.1	95.2	38.6	77.2	33.3	81.8
How CRA drugs are administered^||^ (drug administration in CRA^||^)	41.1	80.2	29	65.5	53.3	92.4	53.7	92.6	40	89.1
Post-CRA care^||^	38.5	37.4	25	32.5	33.3	33.3	52.6	42.1	72.7	54.5

* NT = Nursing Technician;

† INT = Medical Intern;

‡ NUR = Nurse;

§ RES = Medical Residence and Nurse;

|| CRA = Cardiorespiratory arrest;

¶ CRR = Cardiorespiratory resuscitation;

** BLS = Basic life support;

†† ALSC = Advanced life support in cardiology

In the EDS results, the mean scores of the participants’ agreement levels in relation to the objectives and information, support, problem solving, feedback/reflection and realism ranged from 4.34 to 4.79 (from a possible score of 1 to 5), with the highest score being feedback/reflection (4.66) followed by support (4.60) (Table 3).

**Table 3:** Descriptive statistics for the items of the Simulation Design Scale, n=91. Cuiabá, MT, Brazil, 2018

	Items	MN*	SD^†^	p^‡^
Factor 1 - Objectives and information				
	1. At the start of the simulation, enough information was furnished to provide guidance and encouragement	4.34	0.83	0.901
	2. I clearly understood the purpose and objectives of the simulation	4.6	0.53	0.896
	3. The simulation provides enough information, clearly, for me to solve the problem situation	4.43	0.72	0.895
	4. Sufficient information was provided during the simulation	4.45	0.64	0.894
	5. The clues were adequate and directed to promote my understanding	4.51	0.69	0.896
	Factor 1 overall	4.47	0.69	
Factor 2 - Support				
	6. Support was provided in a timely manner	4.55	0.67	0.897
	7. My need for help was recognized	4.59	0.52	0.895
	8. I felt supported by the professor during the simulation	4.58	0.63	0.895
	9. I was supported in the learning process	4.69	0.51	0.894
	Factor 2 overall	4.6	0.59	4
Factor 3 - Troubleshooting				
	10. Autonomous troubleshooting was made easier	4.47	0.58	0.901
	11. I was encouraged to explore all the possibilities of the simulation	4.51	0.6	0.899
	12. The simulation was designed for my specific level of knowledge and skills	4.46	0.73	0.898
	13. The simulation allowed me the opportunity to prioritize nursing assessments and care	4.55	0.6	0.902
	14. The simulation provided me with an opportunity to set goals for the care of my patient	4.69	0.49	0.899
	Factor 3 overall	4.54	0.61	
Factor 4 - Feedback/Reflection				
	15. The feedback provided was constructive	4.77	0.42	0.898
	16. The feedback was provided in a timely manner	4.64	0.55	0.899
	17. The simulation allowed me to analyze my own behavior and actions	4.79	0.41	0.902
	18. After the simulation, there was an opportunity to obtain information/feedback from the professor in order to build up knowledge to another level	4.45	0.7	0.901
	Factor 4 overall	4.66	0.55	
Factor 5 - Realism				
	19. The setting resembled a real-life situation	4.4	0.7	0.899
	20. Real-life factors, situations and variables were incorporated into the simulation setting	4.49	0.64	0.899
	Factor 5 overall	4.45	0.67	
Overall Scale				
	Overall of all items	4.55	0.63	0.898

* MN = Mean;

† SD = Standard deviation;

‡ p = p value

## Discussion

The results of this study demonstrated that teaching CRR to a multi-professional team of a university hospital by HS resulted in a significant increase in the knowledge and ability of the participants to perform the procedures, as well as in high agreement of their satisfactory answers to items related to the structure and methodology of the simulation technique used. Although the participants had previously worked in CRA, the great majority reported never having participated in courses in the theoretical-practical immersion modality. They had only participated in some educational action involving the theme of basic or advanced life support. A study conducted in 2009 related the high cost of immersion courses as one of the factors that may influence the low adherence of health professionals to courses in this modality^19^. 

In addition, the lack of time and obligation of the institutions where they operate and the lack of courses offered in the region where the study was developed are also limiting factors for adherence. This situation is worrying for the managers, and shows a relevant demand for training, since the performance in CRA requires from the rescue team clinical skills and abilities, as well as assertiveness in decision-making, to positively impact on patient survival^18^. 

Resuscitation teams should essentially have standardized care, in which each team member knows his or her role, to ensure a more efficient and faster response pattern^18^. Such peculiarities require teaching methods that result in changes in practices and in the acquisition of multiple skills that, although not complex, require high levels of attention, communication and proactivity from the professionals. Undoubtedly, HS is an adequate method for this type of teaching, considering the characteristics of leading the participant to realistically experience problem situations of this nature^2^^,^ 5. 

Throughout its evolution, the mode of professional training in the health context is based on specific activities, restricted to the performance of training, mostly conducted in a manner disconnected from the reality experienced by professionals, and their knowledge gaps^20^. 

Among the analyzed categories, the nursing technicians obtained greater increase in the percentage of improvement in the cognitive assessment, when compared to the interns, nurses and residents. Other studies^19^^,^ 21 justify that this better performance can be related to the lesser level of scientific knowledge of the technicians in relation to the other categories and attests the impact of the HS on their acquisition of competences. On the other hand, the participants with a higher degree of training obtained a higher number of correct answers in the assessments. The research results reinforce that the higher the level of training, the better the rate of correct answers in cognitive assessments^21^. 

The expected performance in cognitive assessments was equal to or higher than 70%. However, before the intervention, the participants’ knowledge was below the expected for all categories, which reinforces the importance of the use of HS in the acquisition of knowledge, because effective actions in relation to CRA require relevant theoretical and practical knowledge. This finding is congruent with a research study conducted in Israel, which assessed the knowledge of 185 physicians about BLS and concluded that they were not prepared to initiate life-saving procedures^22^. Therefore, finding ways to motivate the participation of the health professionals and using innovative and effective methods for teaching related to the theme of CRR can be good alternatives to improve this outcome. 

Regarding the critical items necessary for CRA care, the proper handling of electro-therapy is cited as one of the crucial procedures for patient survival. The third link in the survival chain is early defibrillation, reinforcing the importance that those involved in this type of care be able to handle electrical therapy equipment^18^. It was identified in this study that there was a low increase in the mean of correct answers in the pre- and post-assessments, in relation to the handling of the manual external defibrillator. This result can be related to the few, even nonexistent, previous training actions related to the handling of the manual external defibrillator cardioversor (MEDC), in the health area courses and in the activities of the permanent education programs at the hospital institution. 

Another fact that may have influenced this result is the existence of more than five brands of MEDC, which makes it difficult to fix the learning about handling. In many CRA cases, electro-therapy is the only therapeutic resource indicated for the treatment and possible reversal of the condition. The medical professional is responsible for indicating this type of therapy in conventional defibrillators; however, a team of rescuers trained in this conduct can contribute effectively in decision-making. 

A study that evaluated the knowledge of the multi-professional health care team in relation to emergency care in the face of a cardiorespiratory arrest, based on the AHA guidelines of 2010^23^, evidenced insufficient knowledge of the professionals to care for CRR, and reinforced the need for investments in updating courses and frequent assessments of professionals in service, since academic papers prior to the publication of the protocol already pointed to similar results. 

In this context, it is understood that more investments should be implemented in health training, and that converge to interventions similar to this study, since there is evidence in literature^24^ that address the inability of academics to perform specific techniques in emergency situations, revealing the distance of knowledge learned and its application in practice. 

Early defibrillation is highly recommended in the survival chain of patients, being the 4th link in the intra-hospital survival chain and the 3rd in the out-of-hospital one^18^. Although HS has had an important impact in changing knowledge about this critical item, more actions mediated by HS, with emphasis on electrical therapy, can be implemented to improve the indicator obtained in this study. 

The team’s low previous knowledge of airway management in CRA by all the professionals reinforced the importance of the training undertaken, as this is a skill that can be delegated to any of the categories participating in the study. Although this knowledge increased with HS, the observed result was still unsatisfactory, i.e., below 70%. Similarly, pre-intervention assessments showed a low level of knowledge about the ability to correctly administer the drugs indicated in CRA and the correct technique of chest compression, which improved substantially after HS. This finding shows the importance of the intervention applied by HS, since drug therapy is fundamental in the management of CRA in patients with non-shockable cardiac rhythms^18^.

Regarding drug administration in CRA, results were 41.1 in the pre-test and 80.2 in the post-test. Regardless of an improvement in this result, the low score at the time before the intervention is considered, knowing that drug therapy is fundamental in CRA management^18^. 

The resourcefulness of the participants in the external chest compression technique is questionable, as it is one of the most discussed contents when the subject is basic and advanced life support, and even so, the results before the intervention were unsatisfactory. Immediate care to CRA is essential because it is a situation of extreme urgency, in which the correct application of the CRR maneuvers is crucial for its reversal^18^. 

Another important result of the study was the unsatisfactory score regarding the diagnostic recognition of a CRA. In the case of nurses and nursing technicians, who are the closest professionals to a patient in CRA, this becomes even more serious. The recognition of a CRA is the main key to trigger help from rescuers in support, as well as to start event modifying care^18^. Fast and effective decision-making depends a lot on this ability, even more so when it comes to grievances that, with each minute that passes, hinder resuscitation success. 

As for the participant’s successes in performing the CRR sequence in BLS, it was satisfactory only after performing the HS. The insertion of BLS as a mandatory activity in the curricula of the health courses can be an important action to increase the quality of care for CRA by students^25^. The still very present use of teaching methods that are not guided by practical activities and active methodologies can justify the lack of assimilation of content such as care for a CRA victim. The study demonstrated HS as one of the most effective alternatives in its training^26^^-^ 28. The increase of confidence, due to the increase of technical knowledge and potentiated by the use of HS, contributes to the participants of this type of activity to have more safety to act in the face of a CRA^29^.

In the health context, a simulated setting can be understood as the reproduction of a clinical situation that provides the development of specific learning objectives of a professional practice^2^. This type of teaching-learning methodology requires well planned and structured criteria, adequate methods, and trained professionals with sufficient physical and human resources to achieve the established objectives^2^. It is important to have authenticity of the simulated environment, when the objective of the training is the substitution of the real clinical environment. The elements of the actual clinical environment must be within the setting and the participants need to identify and perceive them represented during the simulations^6^.

Assessing satisfaction through ESD agreement levels allows building a foundation for improvement and building a portfolio of simulated cases to be implemented, for the academic community and the health care network^3^. In the present study, the scores of the items evaluated in the ESD were between 4.45 and 4.66. This indicates that satisfaction with the information provided at that time was high. The ESD for care training in clinical emergency situations has been one of the most indicated teaching-learning methods, and can be mixed between expository classes, e-learning, and monitored practices^7^.

Another factor of the scale with a high score (4.47) was in relation to the objectives and information provided before and during the simulation. These results are in line with the following works: 3.60^30^, 4,52^31^, 4.40^32^, 4.08^13^ and 4.01^33^. These actions are crucial for assessment, since confidentiality contracts are signed for activities, information related to the clinical case (clinical history, tasks to be performed), who acts, that is, everything that corroborates the participant’s insertion in the setting^6^.

The offer of support to the participants of a simulated activity is also fundamental for the best use and engagement at the climax of the simulation. This item had a mean of 4.60, demonstrating success in the HS method used. Other studies found similar results^13^^,^ 31 ^-^ 33. The role of the facilitator is to ensure that the participants do not leave the same as they arrived for the simulation, i.e., that they can positively impact on the experience of reflexive thinking of their actions, encouraging clinical judgment, enabling improvements in their performance, satisfaction and self-confidence so that transformations are incorporated into their care practice, guided by humanity and efficiency^6^. 

Related to problem solving, the role of the facilitator in stimulating the autonomy of the participants in solving problem situations disposed to them, planning them with the appropriate level of complexity, is highlighted. Thus, the present study reached a mean of 4.54 regarding problem solving, which refers to a level of agreement equivalent to other studies^13^^,^ 31 ^-^ 33. The simulators and the simulation environment can also be key to encouraging problem solving by participants^4^. 

The setting item with the highest score was feedback/reflection, with a mean of 4.66. The participants believe that the feedback provided was constructive (4.77), provided in due time (4.64), that the simulation allowed for the analysis of the behavior and actions themselves (4.79), and that after the simulation, there was opportunity to obtain information/feedback from the professor, in order to build up knowledge to another level (4.45). These results are similar to those of other studies^16^^,^ 31 ^-^ 32 which had means between 4.73 and 4.86. It is known that the feedback/reflection factor is very important in the HS process, because it matches the debriefing moment, where the educational practice is signified and transported to the professional activity or real life of each participant. 

Realism showed high results in the psychometric items. It is known that more important than the technological and structural resource, as high value simulators, is the HS method applied in an adequate manner, based on good simulation practices. The use of equipment of brands and models already used in the institution can also be related to the high satisfaction with the realism experienced by the implemented HS. However, the better structured the HS, the better will be its capacity to produce a positive impact, such as changing professional practices, indicators and behaviors at professional, personal and institutional levels^5^. 

There are important factors to be considered in the degree of realism, namely: physical, environmental and emotional. However, the excess of props can be considered a serious failure in the construction of settings, since it can contribute to the increase of artificialism, which can result in the participant blocking activities^7^. Furthermore, the culture of patient safety, enhanced by the improvement of the simulation centers, should be prioritized in the insertion of the pedagogical planning of subjects in professional training courses, as well as in proposals for continuing education in health environments^34^.

Another factor that can be related to the score of realism obtained is the multi-professional work implemented. The fact that each participant can act in his or her role reinforces the importance of his or her role in relation to a CRA. Multi-professional team building activities are not usual in the Brazilian context^23^, which strengthens the importance of carrying out this study, especially for the institution under study, since it covered several categories of professionals and students involved with the HU. In the practice, it is likely that the results of the HS have favored multi-professional work and enabled the necessary interfaces to good teamwork practices. 

Another potentiality of this study is the integration between teaching and service in the same organizational environment, which can produce changes in the current practices of training and health care. In the context investigated, this stands out with the use of a hybrid simulation, which was able to replicate real life aspects of the involved subjects. 

Among the limitations of the study we can highlight the absence of a comparison group, which directed the study to a before and after type of research, which may not reflect the real effectiveness of the method, since the effect of the background of each participant as co-responsible for their learning performance cannot be ruled out. Another point was the non-random sampling of the research participants, which can limit the generalization of the results.

## Conclusion

This study showed a statistically significant increase in knowledge, with an increase in cognitive learning of 81.9%, especially for the NTs, which was 117.8%. Above all, it was concluded that the theoretical knowledge in CRA/CRR was insufficient, through the mean of the correct answers of the participants in the pre- and post-test.

The HS performed presented an excellent level of satisfaction in relation to the design of the applied setting. The quality of the debriefing, a key-moment of an HS, had the best result, indicating the quality and high level of satisfaction for the participants in the study. 

Through these results, the proposed objective is accomplished and the need for new research studies using health simulation is evidenced, especially to enable concrete contributions to safe and quality practice in the health services, and to scientifically advance the production of knowledge.
